# Competency building for lay health workers is an intangible force driving basic public health services in Southwest China

**DOI:** 10.1186/s12913-019-4433-2

**Published:** 2019-08-23

**Authors:** Shengxiang Liang, Haoyue Deng, Shili Liu, Geng Wang, Li Li, Mei Wang, Jie Pu, Wei Xing, Xingneng Luo, John Ehiri, Yueying Xiang, Ying Li

**Affiliations:** 1 Department of Social Medicine and Health Service Management, Army Medical University (Third Military Medical University), No.30 Gaotanyan Road, Shapingba district, Chongqing, 400038 China; 2 Department of TB control, Center of Disease Control in Shapingba District, Chongqing, China; 3 Division of Health Promotion Sciences, Mel & Enid Zuckerman College of Public Health University of Arizona, Tucson, AZ USA

**Keywords:** Basic public health service, Lay health workers, Competency building, Training

## Abstract

**Background:**

Providing universal basic public health services (BPHS) for residents is the main goal of the new health reform in China. Lay health workers (LHWs) in primary health care (PHC) sectors play key roles in BPHS delivery. The competency of LHWs is critical to quality BPHS. This study assessed LHWs’ competency to deliver BPHS and related training in resource-limited Southwest China.

**Methods:**

A mixed research method combining in-depth interviews with secondary data collection was used to collect data in this cross-sectional study. Fifty-four LHWs and 16 leaders in 16 PHC sectors were recruited for in-depth interviews. Secondary data on 198 LHWs were collected through standard forms.

**Results:**

Both the interviews and secondary data suggested that all PHC sectors did not have sufficient LHWs and lacked qualified LHWs to deliver BPHS overall, particularly in relatively low economic rural areas in Guizhou province. Furthermore, PHC sectors had difficulties retaining existing LHWs due to low incomes and fewer opportunities for self-development. In-depth interviews discovered that, although numerous training opportunities have been provided for LHWs since 2009, the trainings did not achieve the expected outcome in LHW competency building, as LHWs actually did not have access to the trainings and the training design was unresponsive to the actual needs of LHWs. Both LHWs and leaders expressed an urgent need for effective training for LHWs based on systematic needs assessments and the use of qualified trainers and materials.

**Conclusions:**

The shortage of qualified LHWs in PHC sectors became the bottleneck for BPHS delivery in Southwest China. Recent trainings for LHWs were less effective with regard to LHW competency building. A need-based professional training programme for LHWs by qualified trainers was expected by both LHWs and leaders in PHC sectors.

**Electronic supplementary material:**

The online version of this article (10.1186/s12913-019-4433-2) contains supplementary material, which is available to authorized users.

## Background

The emergence of health inequity is a prominent public health issue worldwide, and tackling health inequity has increasingly become a global priority [1]. In April 2009, China implemented her ambitious new health care reform plan. The goal of the reform is to provide affordable and equitable basic health care for all residents by 2020. One of the priorities of this reform is the provision of a package of basic public health services (BPHS) for all residents, thereby promoting the gradual equalization of BPHS among all urban and rural communities [2]. Since 2009, national BPHS programmes have been widely carried out across primary health care (PHC) sectors in China, which plays an important role in ensuring and improving the health condition of residents, and the equity and accessibility of public health have greatly improved [3, 4]. Since 2016, the BPHS package includes 12 categories (health records management for residents, health education, vaccination, health management for children ages 0 to 6 years, maternal health management, health management for the elderly, health management for patients with hypertension or type 2 diabetes, health management for people with severe mental illness, health management for patients with tuberculosis, health management of Chinese traditional medicine, reporting of infectious diseases and public health emergencies, and health administrative oversight) and 48 items [5]. BPHS covered public health services for all residents and for some special populations, such as children, pregnant women, the elderly, and patients with chronic diseases or severe mental illness [5].

The majority of BPHS programmes are delivered by lay health workers (LHWs) in PHC sectors in China [6]. LHW in China refers to the health worker who works in PHC sectors. They have formal professional (general practitioners and nurses) or paraprofessional tertiary education (such as college), whose roles include providing BPHS or basic clinical medicine to community residents, which are important services for improving health equity [7]. PHC sectors in China consist of community health centres (CHCs) and community health service stations (CHSSs) in urban areas and township hospital centres (THCs) and village clinics (VCs) in rural areas [8]. In China, due to the shortage of LHWs in PHC sectors, except for a few general practitioners and public health professionals, most of the LHWs engaged in BPHS used to be nurses and medical technicians, and some do not even have a formal medical education background [9]. Therefore, the Chinese government increased funding and infrastructure for PHC, as human resources for health (HRH) in both quantity and quality are crucial to health services [10], and many trainings have been provided for LHWs. However, a shortage of competent LHWs in PHC sectors continues to be remarkable [11–14]. It has been previously demonstrated that building the capacity of PHC sectors and LHWs’ competency is an important strategy for the success of BPHS [15]. In 2012, the Department of Maternal and Child Health Care and Community Health of Ministry of Health of PRC issued *the Notification of 2011 Major Special Training for Health Workers of Medical Reform* [16] and *Guidelines for implementation of capacity building of Community human resource* [17], which provided guidelines for the training of LHWs in PHC sectors. At present, LHW training is a traditional face-to-face training by trainers from hospitals, medical universities, specialized public health institutions (CDC, etc.) or PHC sectors and is organized by the Health and Family Planning Commission (HFPC) at all levels; the training mainly focuses on polices and service specifications for BPHS [17].

Although the implementation of training began a few years ago, PHC sectors across the country are still confronting low LHW competence [11, 18, 19], which suggests the lack of effective training. Thus, it is critical to assess current LHW training to determine which factors contribute to current poor training outcomes. Such an investigation would provide objective data to develop an evidence-based effective competency building programme to further improve of the quality of BPHS. Few studies about local training programmes in Guangdong and Zhejiang provinces (where better economic conditions are in place) disclose that inadequate and less effective training was provided to LHWs in PHC sectors [20, 21].

Consistent with relatively poor social and economic development in southwest China, PHC sector development lags far behind that of eastern and central China. Although health resource development in PHCs in western China has recently achieved progress, there is a large gap in LHWs between western, central and eastern China [22, 23]. Based on the information obtained from Chongqing [24], there are some existing problems in LHW training, but evidence is required to inform the development of training programmes. This study applied mixed research methods to assess LHW competency in delivering BPHS and the current training programme to determine the existing problems in LHW training and assess the training needs to provide evidence for training programme improvement.

## Methods

This cross-sectional survey utilized mixed research methods to collect data from August 2015 through January 2016 in the PHC sectors of southwest China.

### Study setting

Since the implementation of the “Development of Western China” strategy by the Chinese central government in 1999, western China has become one of the fastest growing economic areas in China [25]. During the 12th Five-Year Plan (FYP) for 2012–2015, the GDP growth rate was 12.24% in 2015 [26]. However, western China still lags behind central and eastern China in socio-economic development. The western China region is an underdeveloped region with substantially lower per capita net income than those of central and eastern China.

A multi-stage randomized sampling method was utilized for selecting study locations in southwest China. Chongqing Municipality (a developed socio-economic region) and Guizhou province (a less developed socio-economic region) were selected for the current study.

One representative socio-economic development county from Guizhou and one representative district from Chongqing were selected. PHC sectors were divided in selected counties/districts into THCs in rural areas and CHCs in urban areas. THCs and CHCs were grouped into developed or less developed regions based on local BPHS reports in previous years. Then, 2 THCs and 2 CHCs from each level were randomly selected. In total, 8 THCs and 8 CHCs were chosen as the final study sites.

### Demographical data collection

Demographical data of LHWs for BPHS from all 16 selected PHC sectors during the study period including gender, age, education level, academic major and professional title, were collected through a form developed by the researchers. Directors of the department of BPHS in all PHC sectors were contacted and facilitated our data collection.

#### Qualitative research

In-depth interviews were performed with all LHWs who delivered BPHS and leaders who were responsible for the BPHS programme in the study PHC sectors. During recruitment, all LHWs who deliver BPHS and leaders who are responsible for the BPHS programme in the study PHC sectors were approached and provided with detailed explanations about the study and its objectives. Those who expressed interest in volunteering to participate in the in-depth interview were asked to read the informed consent form and then were asked to sign the informed consent form as a confirmation of their voluntary participation in the study. In-depth interviews were facilitated by semi-structured topic guides. The topic guide for leaders (see Additional file 1) mainly elicited information about their perspectives on the human resource situation for BPHS (i.e., the number of LHWs delivering BPHS in their own PHC sector, the quality of those LHWs and the stability of LHWs), perspectives on the current LHW training programme, problems with the current training programme, and the need for further training. The topic guides for LHWs (see Additional file 2) included their self-assessment of their competency to deliver BPHS, their participation in training, their perspective on the current training, and the need for further training.

All interviews were conducted in the local language in meeting rooms of PHC sectors or LHWs’ offices. The senior researchers (SLiang, SLiu, LL, YL) conducted the interviews. Each interview lasted approximately 40–60 min. All the interviews were audio-recorded with the consent of the participants.

### Data analysis

#### Quantitative analysis

Epi Data 3.1 was used to enter record data. The record data were analysed using the Statistical Package for Social Science (SPSS 21.0). A two-tailed probability level of *p* < 0.05 was chosen as the level of statistical significance. Missing data were excluded from the analysis. Of the proportions of LHWs by gender, age, education level, academic major and professional title were expressed as percentages. The differences in gender, age, education level, academic major and professional title between rural and urban areas; the differences in the quality of PHC (between developed and less developed); and the differences between regions (Chongqing and Guizhou) were determined by adopting the chi-square test (*p* < 0.05).

#### Qualitative analysis

All the data from interviews were analysed using the framework approach [27, 28] following a five-step process: familiarizing, indexing each transcript with a framework, summarizing data in an analytical framework, data synthesis and the interpretation of the data [27, 29]. First, all the interviews (audio recordings of the interviews and notes) were transcribed into Word documents in mandarin. Second, a coding framework was devised based on a topic guide, and familiarizing transcripts and transcripts were coded with the coding framework. Third, themes were derived from the coded text. Regarding the human resources situation in PHC sectors, themes included the adequacy of LHWs, post provided by the government, education level, academic major and professional title, and the competency and stability of LHWs. Themes on training included training opportunity, training organization institution and trainer, training materials and content, training approaches and training period. There were three subthemes under each theme on training: status, problems and needs. Fourth, all the transcripts were classified with themes and subthemes. The names of all the participants in the in-depth interviews were removed from the quotations in the results to maintain their anonymity.

## Results

### Characteristics of participants

The demographic characteristics of the participants interviewed are presented in Table 1. Sixteen leaders and 54 LHWs from 16 PHC sectors were included in the in-depth interviews. Among the leaders, 8 were from the CHCs and 8 were from the THCs; nearly two-thirds of the leaders (10/16) were female; more than half of the leaders (9/16) were between the ages of 30 to 40 years; three-fourths of the leaders (12/16) had worked for more than three years in the region. Among the LHWs, more than half (29/54) were from CHCs, and the others (25/54) were from THCs; more than three-fourths (42/54) were female; the vast majority of LHWs (49/54) were aged ≤40 years. The majority of LHWs (33/54) had worked less than 3 years in the region.

**Table 1:** Demographic characteristics of the LHWs interviewed (*n* = 70)

Characteristics	Leaders	Frontline LHWs
Gender		
Male	6	12
female	10	42
Age		
< 30	0	20
30–40	9	29
> 40	7	5
Year of working		
< 1	0	8
1–3	4	25
≥ 3	12	21
PHC sector		
CHCs	8	29
THCs	8	25

Notes: LHWs refer to lay health workers; PHC refers to primary health care; CHCs refer to community health centres; THCs refer to township hospital centres

### LHWs for BPHS in PHC sectors

First, most of the LHWs (56/60) had often undertaken more than one BPHS programme, and all the LHWs had heavy workloads (Table 2). All the leaders believed the PHC sectors did not have sufficient LHWs for BPHS, particularly general practitioners and public health professionals (Table 2).

**Table 2:** Capacity of LHWs delivering BPHS in PHC sectors

Themes	Results	Sample of illustrative quotes
Staffing	Almost all the LHWs reported that they had to undertake several BPHS programmes simultaneously. The workload to deliver HR and health management for residents is very heavy. Almost all leasers revealed that PHC sectors lacked staff to deliver BPHS, particularly general practitioners (GP) and public health professionals.	Indeed, there is a lack of personnel…, everyone has a lot of work to do, at least two or three projects at hand. (Director of BPHS department in Chongqing PHC sector) Our staff is definitely not enough. We 7 staff deliver HR for more than 50,000 residents. (LHW responsible for Health records) All THCs lack public health professionals, and almost all BPHS programmes are delivered by nurses. (Director of PHC sector in Chongqing)
Staff size	All the leaders in the PHC sectors consistently reported inadequate posts for BPHS provided by the government in PHC sectors.	This THC does not have enough staff …. For example, we have a population of 100,000 in this township, but at the present, the government only provides 25 posts in this TCH for BPHS delivery, which cannot meet the requirements of the public health service. (Director of PHC sector in Chongqing)
Stability	Nearly all the leaders consistently agreed that LHWs were unstable due to low income and limited personal development.	We have 7 staff in the Department of Public Health, and all of them are retired nurses. They are not stable due to the low salary, and they often resign if they get new job. (Director of PHC sector in Guizhou) The staff in the Department of Public Health is indeed not stable. Within 2 to 3 years, all of the staff turnover. (Director of BPHS department in Chongqing) People are not willing to work in PHC sectors because the salary is too low there. That is the first reason. The second reason is due to the limited career development for young people. There are very few opportunities for promotion to a high professional title, which is a disadvantage of personal development for the youth. (Director of PHC sector in Chongqing)
Academic background structure	A majority of the leaders reported that the majority of LHWs had low levels of education, college or below.	We have 9 staff members in the Public Health Department, but only one graduated from college. …. You can see, the majority were nurse and graduated from technical secondary schools. We even have difficulty recruiting a clinic doctor who graduated from a medial university. (Director of PHC sector in Guizhou)
Major structure	All the leaders reported that the PHC sectors lacked staff with backgrounds in public health; most of the LHWs are nurses.	Now we really lack staff. We have no public health professionals. We have nine staff in the Department of Public Health with only one physician …. (Director of PHC sector in Chongqing) In our CHC, now there are 18 staff; only 2 of them are doctors, and the rest are newly recruited nurses. They are only capable of filling out the forms related to BPHS delivery, but they have little knowledge about chronic disease management, such as hypertension, diabetes (Director of PHC sector in Guizhou)
professional title structure	All the leaders remarked that the majority of staff delivering BPHS only have junior professional titles. It is difficult to be promoted to higher titles because limited high professional titles are assigned to PHC sectors by the government, which in turn results in residents’ lower trust in the PHC sector’s quality.	As for professional title, only 3% of staff have the opportunity to be promoted to a high professional title. In this CHC, only 1 staff member can have a high professional title among the 30 staff. There are no more staff with a high professional title; we cannot provide qualified services to residents, and the residents would not trust your skills. (Director of PHC sector in Chongqing)
Competency	Most of the LHWs considered themselves incompetent to undertake their jobs due to a lack of professional knowledge or skills. Almost all the leaders complained that further competency building of LHWs is needed to deliver BPHS, particularly management of patients with severe mental illness and chronic disease management. LHWs lack professional knowledge and skills to guide medicine use, rehabilitation or follow up and assessment.	People with both professional knowledge and communicative skills are rare. (LHW responsible for Chronic diseases management in Guizhou) We lack public health knowledge because most of us were nurses before. Now we are engaged in public health services. We are not qualified. (LHW responsible for health management for the aged in PHC sector in Guizhou) We are incompetent. Regarding the education of our nine staff members in the Public Health Department, the highest education achieved is college, and they do not have any clinical experience. A few of them were nurses before, and some of them had no professional titles. They lack professional knowledge and have little knowledge of hypertension and diabetes management. (Director of PHC sector in Guizhou) We lack professional staff for severe mental disease management,..., and our staff can only provide medication guidance and regular monitoring of the side effects of medicines. (Director of PHC sector in Chongqing)

Notes: BPHS refers to basic public health services; HR refers to health record; PHC refers to primary health care; LHWs refer to lay health workers;

Second, regarding the quality of LHWs, both the LHWs and the leaders complained that all LHWs delivering BPHS were poorly educated (having less than a technical secondary education), had a low professional title (junior), and had little background in public health and/or clinical medicine. Most BPHS programmes were delivered by nurses from the clinical department of PHC sectors. The majority of LHWs felt incompetent in delivering BPHS, which was consistent with the leaders’ reports (Table 2). As presented in Table 3, the results from the secondary data indicated that the percentage of females was apparently higher than that of males (83.8% versus 16.2%) in PHC sectors, particularly in urban PHC sectors (89.4%) and developed PHC sectors (89.7%). Among the LHWs, 86.9% (172) were less than 45 years old, and 38.9% (77) were between 25 and 34 years old. A significant difference was found among the five age groups between PHC sectors in urban Chongqing and rural Guizhou (*P* < 0.05). Among LHWs, 79.3% (157) only had a college education or less, which was consistent with the in-depth interviews. LHWs’ education was better in developed PHC sectors (compared with less developed PHC sectors) and PHC sectors in Chongqing (compared with PHC sectors in Guizhou) (*P* < 0.05). Overall, approximately 50 and 25% of the LHWs had a background in nursing or clinical medicine, respectively. Interestingly, only 6.7% of the LHWs had a public health background, particularly in less developed PHC sectors, and nurses shared 60% (*P* < 0.05). Nearly 80% of the LHWs only had a junior or no professional title, and this percentage was significantly higher in PHC sectors in rural areas, less developed PHC sectors, and in Guizhou (*P* < 0.05).

**Table 3:** Human resource for BPHS in PHC sectors in study places

Characteristics	Total	Rural/urban		Quality of PHC		Region	
		Urban	Rural	developed	less developed	Chongqing	Guizhou
Gender							
Male	32 (16.2)	11 (10.6)	21 (22.3)	22 (21.8)	10 (10.3)	16 (16.3)	16 (16.0)
Female	166 (83.8)	93 (89.4)	73 (77.7)*	79 (78.2)	87 (89.7)*	82 (83.7)	84 (84.0)
Age							
<25	48 (24.2)	18 (17.3)	30 (31.9)	25 (24.8)	23 (23.7)	20 (20.4)	28 (28.0)
25–34	77 (38.9)	45 (43.3)	32 (34.0)	39 (38.6)	38 (39.2)	49 (50.0)	28 (28.0)
35–44	47 (23.7)	24 (23.1)	23 (24.5)	20 (19.8)	27 (27.8)	16 (16.3)	31 (31.0)
45–54	20 (10.1)	11 (10.6)	9 (9.6)	13 (12.9)	7 (7.2)	8 (8.2)	12 (12.0)
55-	6 (3.0)	6 (5.8)	0 (0.0)*	4 (4.0)	2 (2.1)	5 (5.1)	1 (1.0)*
Education							
Undergraduate	41 (20.7)	20 (19.2)	21 (22.3)	29 (28.7)	12 (12.4)	31 (31.6)	10 (10.0)
College	91 (46.0)	54 (51.9)	37 (39.4)	45 (44.6)	46 (47.4)	55 (56.1)	36 (36.0)
Technical Secondary School	63 (31.8)	30 (28.8)	33 (35.1)	24 (23.8)	39 (40.2)	12 (12.2)	51 (51.0)
High School and below	3 (1.5)	0 (0.0)	3 (3.2)	3 (3.0)	0 (0.0)*	0 (0.0)	3 (3.0)*
Major							
Clinic	48 (24.7)	25 (24.8)	23 (24.7)	30 (29.7)	18 (19.4)	21 (22.1)	27 (27.3)
Medical Technician	9 (4.6)	5 (5.0)	4 (4.3)	5 (5.0)	4 (4.3)	5 (5.1)	4 (4.0)
Public Health	13 (6.7)	4 (4.0)	9 (9.7)	6 (5.9)	7 (7.5)	3 (5.3)	10 (10.1)
Health Service Management	6 (3.1)	4 (4.0)	2 (2.2)	6 (5.9)	0 (0.0)	4 (4.2)	2 (2.0)
Nursing	96 (49.5)	53 (52.5)	43 (46.2)	39 (38.6)	57 (61.3)	51 (53.7)	45 (45.5)
Other	22 (11.3)	10 (9.9)	12 (12.9)	15 (14.9)	7 (7.5)*	11 (11.6)	11 (11.1)
Professional Title							
Associate Senior	4 (2.0)	1 (1.0)	3 (3.2)	1 (1.0)	3 (3.1)	4 (4.1)	0 (0.0)
Attending Doctor	20 (10.1)	12 (11.5)	8 (8.5)	12 (11.9)	8 (8.2)	11 (11.2)	9 (9.0)
Junior	113 (57.1)	71 (68.3)	42 (44.7)	66 (65.3)	47 (48.5)	61 (62.2)	52 (52.0)
No	61 (30.8)	20 (19.2)	41 (43.6)*	22 (21.8)	39 (40.2)*	22 (22.4)	39 (39.0)*

Notes: BPHS refers to basic public health services; PHC refers to primary health care; *means *P*<0.05

Third, almost all the leaders interviewed stated that LHWs had lower stability in their PHC sectors mainly due to low incomes and limited self-development in the PHC sectors (such as a lack of opportunities to achieve promotions to a senior professional title).

### LHW training on BPHS delivery

A majority of LHWs thought they improved their skills and knowledge to deliver BPHS to some extent through participating in trainings. The vast majority of leaders complained that the current trainings did not improve the competency of LHWs. Each theme of training, status and problems in the current training was analysed, and training needs were identified through the interviews (Table 4).

**Table 4:** Training for LHWs delivering BPHS in PHC sectors

Themes	Sub-themes	Results	Sample of illustrative quotes
Training opportunity	status	All the LHWs and leaders stated that the CDC, Health and Family Planning Commission (HFPC) and hospitals regularly organized trainings for them.	Generally, the hospital provided professional trainings for us once or twice each year. The institution in charge of the PHC sectors often provided 2–3 trainings to us. (LHW responsible for chronic diseases management in PHC sector in Chongqing) Every year the municipal CDC organizes a professional trainings. There are10–20 professional trainings provided by our CHC. (LHW responsible for health education in PHC sector in Chongqing) Every year the institutions in charge of the PHC sectors at all levels organize regular trainings for each BPHS project. (Director of BPHS department in Chongqing PHC sector)
	problems	Over one third of the LHWs claimed they actually participated less in trainings due to a lack of LHWs to deliver BPHS everyday they were offered training opportunities. Similarly, most of the leaders stated that the heavy workload to deliver BPHS by few LHWs, and so, the PHC sectors had difficulty allowing LHWs to participate in trainings.	I seldom take part in any training because we do not have enough staff to do the work. (LHW responsible for health management for the aged in PHC sector in Chongqing) There is requirement for each PHC sector to arrange for LHWs to participate in trainings. I am not unwilling to have LHWs join trainings. However, I have so few LHWs to deliver BPHS daily, and we cannot complete the work if I arrange for one or two LHWs to participate in trainings. Therefore, although we welcome this kind of training, we can’t have staff take part in the training if participating in training results in no LHW to undertake work. (Director of PHC sector in Chongqing)
	needs	All the LHWs expressed their wish to receive more training opportunities and their hope for effective, fruitful training, such as training about professional knowledge and skills.	We hope that our country could offer some responsive trainings for us every year, such as trainings on health management for the aged, which should be specific about how to guide the aged to have a healthy lifestyle…..(LHW responsible for health management for the aged in PHC sector in Guizhou)
Organizational institution	status	All the leaders reported that HFPCs at all levels organize trainings. Training institutions often include hospitals, medical universities, professional health institutions (CDC) and the PHC sectors themselves.	They (HFPCs) offer specialized trainings on BPHS organized at the municipal level. At least five or six LHWs could participate in it. It is the training on the management of pregnant women this time, and it would be about planned immunity or chronic disease management next time. This is the training at the municipal level. There are two or three trainings at the district level. There may be some trainings from the CDC that are provided for all the public health personnel in the region. If there is any change to deliver BPHS, the hospital in change of our PHC sector would organize trainings for our staff. (Director of PHC sector in Chongqing)
	problems	According to some leaders, current training institutions are not appropriate to deliver training. There is no systematic training and no professional institutions to undertake training regarding patients with severe mental illness and chronic disease management.	I think it is best to send our staff to the professional agencies to receive training. For example, training about patients with severe mental illness in the Mental Health Centre and training on chronic disease management in the CDC. We need practical trainings. It is good for PHC sectors to provide trainings themselves. (Director of PHC sector in Chongqing) As for the current training for public health personnel, such as job-transferring training and cadremen training, it was not implemented very well. What’s more, just short trainings cannot provide much systematic knowledge. (Director of PHC sector in Chongqing)
	needs	Some of the LHWs welcome systematic trainings instead of fragmented ones. Most of the leaders thought trainings on professional projects should be provided by professional institutions.	It is best to systematically organize and provide trainings that can avoid repeated trainings. (LHW responsible for health management for the aged in PHC sector in Guizhou) … Take the management of patients with severe mental illness, for example, this training need professional institution. (Director of BPHS department in Chongqing PHC)
Training content	status	A majority of LHWs thought the current trainings mainly focused on polices and service specifications for BPHS.	The main contents of the trainings are public health service standards, policies, and the specifications to fill out related forms. (LHW responsible for health management for the aged in PHC sector in Guizhou)
	problems	1. Most of the LHWs thought current trainings concentrated on policy interpretation, form filling, and system operation. Some leaders reported that the current trainings were not responsive to needs and did not focus on the core competencies of public health, such as professional knowledge and skills. 2. More than half of the leaders complained there was no needs assessment before trainings, which resulted in trainings that do not meet needs regarding the delivery of BPHS and so do not promote the competencies of LHWs to deliver BPHS.	Ah, …, most trainings focus on how to fill out the form related to HR. I do not think it is of great practical significance. (LHW responsible for chronic diseases management in PHC sector in Chongqing) We need to know what the BPHS projects are, and we need to know how to deliver those projects, such as the methods and techniques. These can be obtained through training, but…. (Director of BPHS department in Chongqing PHC) Some trainings begin with the policy on BPHS and rarely involve professional knowledge. Surely we need to know related policies, but I think that it is more important for us to have related professional knowledge than knowledge about the policies …. The training is not responsive. For example, training in the field of maternity and child care should offer related professional knowledge. The training on hypertension patient management should offer professional knowledge of hypertension. We lack professional knowledge. (Director of PHC sector in Guizhou) Sometimes the trainings at the provincial level did not meet needs. I have attended some of the trainings before. I feel that those trainings are too theoretical and are not based on our actual needs, so they is not very useful. (Director of PHC sector in Guizhou)
	needs	The majority of the LHWs hoped that they could receive more training about professional knowledge and communication skills and that the training would be more systematic and responsive their needs. In addition to professional knowledge and skills, the majority of the leaders added that trainings on service skills were still in need.	There should be more trainings on professional knowledge and skills and communicative skills as well. (LHW responsible for health education in PHC sector in Guizhou) Current trainings mainly focus on policies and requirements. We should not only master the basic knowledge of public health services but also the skills to deliver BPHS. These can be obtained through training, but…. (Director of BPHS department in Chongqing PHC) For them (LHWs to deliver BPHS), it is necessary to master some medical knowledge to do health guidance and to have professional knowledge. Second, LHWs need to communicate with residents when they deliver BPHS, and so, it is very important to have communication skills. (LHW responsible for chronic diseases management in PHC sector in Guizhou) For example, I delivered health education to residents for many years. First, I need professional knowledge; second, communication and coordination skills are very important, which require more training. (Director of BPHS department in Chongqing PHC) I think that it’s necessary to assess training needs to improve training efficiency. (Director of PHC sector in Chongqing)
Training approach	status	All the LHWs indicated that the main training approaches are lectures and meeting that are mostly focused on theory knowledge training, and sometimes, there are some practice demonstration trainings and simulation trainings.	Lectures given by leaders or professionals from the CDC and simulation trainings on how to carry out chronic disease management. (LHW responsible for health management for the aged in PHC sector in Chongqing) The form of the trainings is lectures, from HFPCs or the CDC, or by attending meetings in hospitals. (LHW responsible for chronic diseases management in PHC sector in Guizhou)
	problems	Most of the leaders and the LHWs considered the training more theoretical instead of focusing on practical skills, which resulted poor outcomes.	The only form of training is lectures without any clinical practice. (LHW responsible for health management for the aged in PHC sector in Guizhou) Generally, the main form of trainings lectures on theory. There is little training on practice skills. (Director of PHC sector in Chongqing)
	needs	A large number of the LHWs believed that practice-based training was a key to promoting their competence, such as on-site guidance, field observations, and clinical practice. In terms of theoretical knowledge training, it is a good choice to use a network to disseminate trainings through which LHWs could choose their time and site to receive trainings at their convenience	Training should be based on practice, and this kind of training is useful. Current training through lectures once every 3 months based on theoretical materials had less effect. (LHW responsible for chronic diseases management in PHC sector in Chongqing) It’s a pity that I never attend the trainings because of my busy work. I love studying. I like to study through yishitong, which is a medical information service platform. The doctors on the platform are warm hearted, and I can ask them questions online. (Director of PHC sector in Guizhou)
Training period	status	The majority of the leaders and LHWs reported that most of the current trainings were short, from a few days to a few weeks or to one month at most.	The training is often short, and it usually lasts one week or two weeks, one month at most. (Director of PHC sector in Chongqing) There are 4–5 trainings per year, and each training lasts 2 to 3 days. (LHW responsible for health management for the aged in PHC sector in Guizhou)
	problems	The vast majority of the leaders and LHWs thought the short trainings could not promote LHWs’ professional knowledge and competency.	They (LHWs) often attend the short trainings, and it is hard for them to have systematic learning. (Director of PHC sector in Chongqing)
	needs	The vast majority of the LHWs considered that long-term systematic training was needed to promote job competency instead of fragmented training.	Training time is too short, and it should be arranged based on training content. (LHW responsible for health management for the aged in PHC sector in Guizhou)
Training materials and team	status	Many leaders pointed out the lack of useful training materials, except for the specification of BPHS, and the trainers were physicians from general hospitals, specialized hospitals, the CDC or teachers from medical universities.	At present, the trainers are mainly from general hospitals, specialized hospitals, CDCs and medical universities. The main training materials are the specifications of BPHS. Trainers usually selected their preferred training materials. (Director of PHC sector in Guizhou) .…. Many trainers are from specialized hospitals, and they do not know the requirements to deliver BPHS very well. More often, they talk more about theory. (Director of PHC sector in Chongqing)
	problems	Some leaders reported that there are no qualified trainers or training materials.	For example, the training for new LHWs delivering BPHS is not so good. The training is often short and not systematic. LHWs could just learn how to deliver BPHS during work. There is no good trainers or good training materials to train them about how to deliver BPHS. (Director of PHC sector in Chongqing) There are no fixed trainers. There are no qualified training materials except for the specifications of BPHS. (Director of PHC sector in Guizhou)
	needs	Some leaders believed that qualified training materials and trainers were needed to improve the training effects.	I think we need more investment in training, such as inviting some qualified trainers to give lectures, and developed training materials involving different subjects. We should invite experts to organize trainings. The research on this issue is still lagging behind. (Director of PHC sector in Chongqing)

Notes: BPHS refers to basic public health services; HR refers to health record; PHC refers to primary health care; LHWs refer to lay health workers;

#### Opportunity for LHW training

All the leaders and LHWs acknowledged that there were many training opportunities for LHWs on BPHS. However, > 1/3 of LHWs and a majority of the leaders complained that there were consistently short staffed in PHC sectors, which consequently resulted in PHC sectors being unable to arrange for working LHWs to participate in trainings. Nevertheless, LHWs would like to improve their skills and knowledge to deliver BPHS by attending such trainings.

#### Organizational institution and trainer

The provincial or local Health and Family Planning Commission (HFPC), public health facilities, hospitals, medical colleges/universities, or PHC sectors provided trainings on BPHS. However, the leaders felt that the trainings from such institutions was not well organized, resulting in poorly qualified training. The LHWs were keen to be trained systematically on BPHS, and the leaders expressed that the training should be provided by professionally qualified trainers.

#### Training materials and content

Most of the leaders expected formal training textbooks, which should be generated. The vast majority of LHWs felt that the current trainings often mainly focused on policy and service specifications related to BPHS, which might not be as useful for their professional work. Many of the leaders felt that the trainings failed to address the core competency of delivering BPHS and the actual needs of LHWs because there was no needs assessment before the trainings. Both the leaders and the LHWs would like to be trained about professional knowledge and skills related to BPHS delivery based on a needs assessment.

#### Training approach

Many of the LHWs reported that the current trainings were often provided only through lectures. Both the leaders and the LHWs thought the trainings through lectures mainly addressed theory, but the LHWs would like to have trainings on practical skills through work-based training. Theoretical knowledge training could be achieved through the internet, which can provide greater flexibility to avoid conflicts between working and participating in the trainings.

#### Training period

The vast majority of the leaders and LHWs reported that most of the current trainings were short (between a half of a day to one week) and that there is no systematic training, resulting in lower LHWS competency. Therefore, a vast majority of the LHWs preferred longer and more systematic trainings.

## Discussion

The Chinese PHC sectors have long faced the challenge of inadequate LHWs [30]. Thus, capacity building for the PHC sectors has attracted a great deal of attention from the Chinese government with vigorous support in human resources, funding and equipment to improve the capacity of PHC sectors since 2009. Substantial progress has been achieved in the number of LHWs; for example, LHWs in PHC sectors have increased from 3,282,000 in 2010 to 3,683,000 in 2016 [31]. However, PHC sectors still lack adequately qualified LHWs to provide BPHS for residents across China [32–37]. This is more noticeable in western China, where socio-economic development lags behind that of central and eastern China [22, 38, 39]. The current study also demonstrated that PHC sectors lack LHWs for BPHS in southwest China. Even worse is that many LHWs often undertake more than one BPHS programme. It has been reported that the main reasons for the shortage of LHWs are the heavy workload, poor working conditions, low income, and lack of social security for BPHS delivery in Guizhou province [18]. Furthermore, medical students from universities prefer to stay in large hospitals rather than the PHC sectors in rural communities [40]. This study also revealed that the PHC sectors in the study sites were almost unable to attract medical students who had graduated from university and retain existing LHWs due to low incomes and limited self-development, harsh working conditions and heavy workloads. Critically, the shortage of LHWs must be addressed through policy, planning and the implementation of innovative strategies. The Chinese central government has already gradually increased substantial financial support for regions with financial difficulties [7]. The local government in southwestern China should also provide adequate financial support to employ LHWs for PHC sectors to deliver BPHS based on the population covered by the PHC sectors. In addition, a more attractive policy is advantageous to support LHW development with incentives to attract and retain LHWs.

One important strategy to improve the competency of LHWs is professional training [41], which is a central method for the Chinese government to improve the competency of LHWs. The Chinese Minister of Health issued *Guidelines for the implementation of capacity building of community human resources* in 2012 [17]. However, LHW competency improvement across China remains to be achieved as expected [19, 20, 42, 43]. The competency of LHWs in most PHC sectors in China is still low [18, 20, 44–48]. Our current study revealed that the LHWs in PHC sectors in western China had a low professional title, lacked adequate professional education and lacked an academic background in delivering BPHS. In particular, the state of the quality of LHWs was even more serious in PHC sectors with underdeveloped socio-economic conditions (in rural areas, less developed PHC sectors, and Guizhou province).

A few studies assessed the effect of LHW training in Beijing [49], Zhejiang [21, 50], Xinjiang [19], Hebei [51] and Chongqing [24] and found that the current trainings have low feasibility and limited effects. Some studies found that the reasons for ineffective trainings were because the PHC sectors had difficulty with LHWs attending trainings due to a shortage of LHWs [9, 52, 53]; the trainings had no scientific organization [49, 53, 54] and design [43, 49, 52–55], and the trainings lacked qualified trainers and textbooks [43]. The current study systematically assessed LHW training on BPHS and identified problems in the current trainings in western China. First, LHWs had no time to attend trainings due to their heavy workloads. Second, the trainings themselves had shortcomings: there was no systematic organization of the trainings, resulting in fragmented trainings; the training design, content, approach and period were not based on evidence from needs assessments, which caused the trainings to fail to meet LHWs’ needs regarding BPHS delivery; and there were no qualified trainers and textbooks, which also decreased the training quality.

Meng et al. emphasized that LHW trainings must urgently improve their training approaches, develop training materials and even improve whole training systems [56]. Our data are in line with those of Meng et al., showing that LHWs need systematic training to improve their core competency for BPHS delivery. Furthermore, we believe that it is very important to design high-quality training content and materials because LHWs would like to have professional knowledge, practice skills (such as communication skills) and people-centred concepts instead of just BPHS guidelines. Regarding specific professional knowledge and skills, it is also important to study the core competency of LHWs to deliver BPHS programs, which may serve as important evidence for designing training content. Work-based trainings would be desirable for LHWs based on a needs assessment. When designing the training programme, optimizing appropriate LHW training approaches and periods based on LHW needs should be considered. A study from Uganda proves that hands-on, work-based training is a useful model for strengthening health workforce capacity, allowing participants to return to their places of work after each face-to-face interactive session [55]. Such an approach reinforces the theory with practice by enhancing hands-on learning and also allows the trainees to learn from familiar environments and to address real work-place problems. Similar approaches have been used to strengthen health workforce capacity in several countries, including The Gambia [57], Nicaragua [58] and Liberia [59]. The current study identified that professional trainers (with professional knowledge and rich practice experience) are needed, which is one of important factors associated with training effects [60], although they can be from the CDC, medical university or other health facilities [54]. Finally, the government should further increase human resources for PHC sectors to address the shortage and the difficulty of arranging for LHWs to attend trainings.

### Strengthens and limitations

It has been reported that human resources for BPHS evaluated training programmes for LHWs. Our current study focused on identifying problems in current trainings and assessing the need for LHW capacity building in detail (training content, approach, trainer and textbook). Our data may serve as evidence for improving the design of training programmes. Our current study used a qualitative research method (semi-structured, open-ended individual interviews) to assess the capability of LHWs and perceptions about competency building for LHWs from both LHWs and leaders in PHC sectors. Our data provided sufficient in-depth descriptions of existing problems in current capacity-building programmes for BPHS and the need for further training programmes.

However, there are a number of limitations in the current study. First, more desirable outcomes could be achieved if the organizers of the existing trainings are included in this study to obtain a comprehensive and overall assessment. Second, we used only in-depth interviews to collect data, which might compromise the generalizability of our findings. A questionnaire survey with a representative sample of LHWs will be used in a further study. Third, we were not able to identify core competencies for LHWs to deliver BPHS, which will be researched in our next study.

## Conclusions

This study is an important step towards a better understanding of the human resources for BPHS and competency building for LHWs in PHC sectors in southwest China. Although a tremendous improvement has been achieved in China in the coverage of BPHS over the last several years, our study revealed a substantial shortage of qualified LHWs delivering BPHS in rural regions. The training of LHWs has been carried out across southwest China and has had some impact on LHW competency building in PHC sectors. However, the current study discovered that the existing training programme provided in western China did not achieve the expected outcome for the following reasons: LHWs actually had less opportunity to attend training programme(s) due to heavy workloads; and a lack of needs-based training design, qualified trainers and textbooks decreased the training effect. The preliminary needs assessment in the current study illustrated that several key issues related to training need to be resolved to achieve better training effects, such as a training design based on a systematic needs assessment, trainer capacity building and training materials development.

## Additional files

Additional file 1: The Interview Guide for Leaders. (DOCX 22 kb)

Additional file 2: The Interview Guide for Lay Healthcare Workers. (DOCX 27 kb)

## Data Availability

The datasets generated and/or analysed during the current study are not publicly available to avoid compromising individual privacy. It is difficult to adequately de-identify this type of qualitative data. Data may be made available from the corresponding author based on a reasonable request.

## Abbreviations

BPHS: Basic public health services
CHCs: Community health centres
CHSSs: Community health service stations
HFPC: Health and Family Planning Commission
HR: Health record
HRH: Human resources for health
LHWs: Lay health workers
MOH: Ministry of Health
PHC: Primary health care
THCs: Township hospital centres
VCs: Village clinics
