# Supplementary material for: Competency building for lay health workers is an intangible force driving basic public health services in Southwest China
Source: BMC Health Serv Res. 2019 Aug 23;19:596. doi: 10.1186/s12913-019-4433-2 (PMC6708187; doi:10.1186/s12913-019-4433-2)
Supplement: Supplementary file 1 — The Interview Guide for Leaders. (DOCX 22 kb) [file 12913_2019_4433_MOESM1_ESM.docx]

**The Interview Guide for Leaders in Primary Health Care Sectors**

1. What is the current status of basic public health services delivery?

2. What is the current status of the capacity among lay health workers in your primary health care sectors?

3. How about the training on lay health workers who deliver basic public health services?

4. What are the needs to improve the training for lay health workers who deliver basic public health services?

5. What do you think about the career development of the lay health workers who deliver basic public health services?

6. How do you think of your work as a lay health worker to deliver basic public health services?

7. What specific tasks have you done in improve the capacity of lay health workers in your primary health care sectors?

8. Do you have any suggestions for capacity building for delivering basic public health services?
