# Supplementary material for: Competency building for lay health workers is an intangible force driving basic public health services in Southwest China
Source: BMC Health Serv Res. 2019 Aug 23;19:596. doi: 10.1186/s12913-019-4433-2 (PMC6708187; doi:10.1186/s12913-019-4433-2)
Supplement: Supplementary file 2 — The Interview Guide for Lay Healthcare Workers. (DOCX 27 kb) [file 12913_2019_4433_MOESM2_ESM.docx]

**The Interview Guide for Lay Healthcare Workers in Primary Health Care Sectors**

1. How about the delivery of basic public health services?

2. How do you think of your ability to deliver basic public health services?

3. How about the training on delivering basic public health services?

4. What are the needs to improve the current training for lay health workers who deliver basic public health services?

5. What do you think about your career development?

6. How do you think of your work as a lay health worker to deliver basic public health services?

7. Do you have any suggestions to improve the delivery quality of basic public health services?
